# Reflections on the impact and response to the Peruvian 2017 Coastal El Niño event: Looking to the past to prepare for the future

**DOI:** 10.1371/journal.pone.0290767

**Published:** 2023-09-26

**Authors:** Marisol Yglesias-González, Armando Valdés-Velásquez, Stella M. Hartinger, Ken Takahashi, Guillermo Salvatierra, Rodrigo Velarde, Alvaro Contreras, Hugo Santa María, Marina Romanello, Valerie Paz-Soldán, Juan Bazo, Andrés G. Lescano

**Affiliations:** 1 Clima, Latin American Centre of Excellence for Climate Change and Health, Universidad Peruana Cayetano Heredia, Lima, Peru; 2 EcoSalud, Laboratorio de Ecosalud y Ecología Urbana, Facultad de Ciencias e Ingeniería, Universidad Peruana Cayetano Heredia, Lima, Peru; 3 Swiss Tropical and Public Health Institute, Basel, Switzerland; 4 University of Basel, Basel, Switzerland; 5 Geophysical Institute of Peru, Lima, Peru; 6 Emerge, Emerging Diseases and Climate Change Research Unit, School of Public Health and Administration, Universidad Peruana Cayetano Heredia, Lima, Peru; 7 Boston University, Boston, Massachusetts, United States of America; 8 APOYO Consultoría, Lima, Peru; 9 University College London, London, United Kingdom; 10 Health Office of Latin America, School of Public Health and Tropical Medicine, Tulane University, Lima, Peru; 11 Red Cross Red Crescent Climate Centre, The Hague, Netherlands; Universidade de Aveiro, PORTUGAL

## Abstract

Climate-related phenomena in Peru have been slowly but continuously changing in recent years beyond historical variability. These include sea surface temperature increases, irregular precipitation patterns and reduction of glacier-covered areas. In addition, climate scenarios show amplification in rainfall variability related to the warmer conditions associated with El Niño events. Extreme weather can affect human health, increase shocks and stresses to the health systems, and cause large economic losses. In this article, we study the characteristics of El Niño events in Peru, its health and economic impacts and we discuss government preparedness for this kind of event, identify gaps in response, and provide evidence to inform adequate planning for future events and mitigating impacts on highly vulnerable regions and populations. This is the first case study to review the impact of a Coastal El Niño event on Peru’s economy, public health, and governance. The 2017 event was the third strongest El Niño event according to literature, in terms of precipitation and river flooding and caused important economic losses and health impacts. At a national level, these findings expose a need for careful consideration of the potential limitations of policies linked to disaster prevention and preparedness when dealing with El Niño events. El Niño-related policies should be based on local-level risk analysis and efficient preparedness measures in the face of emergencies.

## Introduction

El Nino-Southern Oscillation (ENSO) is a global-scale climate variability phenomenon. It is caused by atmosphere-ocean interactions in which the tropical atmosphere responds to fluctuating ocean temperatures in the equatorial Pacific Ocean. ENSO events occur on a timescale of 3 to 7 years [1], which affects the global climate through atmospheric teleconnections [2, 3]. The warm and cool ocean phases are called El Niño and La Niña, respectively, while the Southern Oscillation is the large-scale atmospheric component of this interaction.

In 1983 and 1998, ocean warming from the coast of South America to the central equatorial Pacific developed into extreme El Niño events and developed according to well-established ENSO mechanisms, albeit with enhanced ocean-atmosphere feedbacks associated with westerly winds along the equator [4, 5]. These elements allowed for prediction several months in advance [6]. In contrast, the onset of the 2017 coastal El Niño event was much more abrupt, with warming of around 7–9°C at the coast over 1–2 weeks [7, 8]. Coastal El Niño events are associated with fast ocean-atmosphere feedback mechanisms restricted to the far-eastern Pacific, involving northerly winds [7–11]. Global climate models could not adequately predict the event, even with a one-month lead time [8, 10].

In general, ENSO events have wide-ranging impacts on global climate conditions and the frequency of hydro-meteorological anomalies, such as floods and droughts [12]. Consequently, ENSOs can be associated with considerable public health impacts, as they can cause increases in the incidence of vector-borne diseases such as dengue, yellow fever and malaria. Moreover, ENSO can reduce the continuity of care due to health infrastructure damage and can affect food insecurity due to adverse effects on agricultural systems and fisheries. Changing rainfall patterns and increasing temperatures on crop yields and water, impact food systems, transportation, market access and other deleterious effects on agriculture and artisanal fisheries [13–16]. Possible damage to public infrastructure or population displacement can also be associated with ENSOs, leading to additional socioeconomic impacts [14, 17, 18].

In recent years, the scientific community has started focusing on the diversity among ENSO events, particularly concerning the spatial distribution and strength of the warming or cooling patterns in the equatorial Pacific [19], as these can determine the resultant climatic anomalies around the world and also. For instance, El Niño warming in the eastern equatorial Pacific enhances precipitation along the coast of Peru, while warming in the central equatorial Pacific tends to reduce precipitation in the Andes and Amazon region [16, 20, 21].

Very intense and recurrent rainfall on Peru’s northern and central coast was observed in 2017. Above-average precipitation led to increases in seasonal river flows, with up to a 50-fold increase compared to historical averages in the Rimac basin located in Lima, and with seasonal river flows above 250% of the average in several other basins and resulted in landslides along the whole length of the Peruvian coast. The event was categorised as a “Coastal” El Niño due to the anomalies in the SST in Zones 1+2 (quadrant that covers the northern part of the Peruvian sea) having its effects on the Pacific coast of South America [22].

The 2017 measured precipitation and subsequent increase in river flows had a significant impact on the health and socioeconomic wellbeing of an estimated 1.9 million Peruvians (approx. 6% of the population) [22–25]. It caused 169 deaths and 18 missing persons, displaced 194,000 people, and damaged more than three hundred thousand homes while leaving 65,000 homes uninhabitable [26]. Significant economic losses were attributed to this and other public infrastructure damage, such as the collapse of water distribution systems and associated drinking water shortages [27, 28].

We describe relevant climatic conditions during the Coastal El Niño 2017, differentiating the development and impacts of that event from those of the well-known extreme global-scale El Niño events of 1982–1983 and 1997–1998, which featured the strongest warming in the far eastern Pacific since 1900, far exceeding other events in terms of the warming strength in this region [29]. On the other hand, Takahashi & Martínez (2017) [7], using different indicators of the hydroclimate of coastal Peru, showed that these two events and the 1925 coastal El Niño were the strongest El Niño events between 1910 and 2000, while Peng et al. (2019) [10] showed that the 2017 coastal El Niño event was the strongest in terms of the far eastern Pacific SST and precipitation after 1983 and 1998. Note that even though the 2015–16 event is generally considered to be a strong El Niño, it did not enhance coastal precipitations [6] and can be classified as moderate in terms of its eastern Pacific signature [30]. Thus, in terms of their hydroclimatic manifestations, we focus on 1925, 1983, 1998 and 2017 as the strongest El Niño events along the coast of Peru since the early XX century.

Using publicly available climate, health, and economic data, as well as historical literature on global ENSO events, we also assessed the national and sub-national economic impacts of Peru’s 2017 Coastal El Niño, described, and compared the epidemiological data and recorded rates of mandatory-reporting infectious diseases, and analysed the associated government preparedness and response during the event. Lessons learned from the 2017 Coastal El Niño can provide a good basis for better preparedness and responses for future events.

The analysis and findings of this study are relevant for broader disaster and risk management in Peru. Considering that beyond El Niño events, scenarios of heavy rains and floods linked to a changing climate are also expected, this case study aims to highlight the urgency of adopting preparedness and response measures to prevent future impacts on Peru’s public health and the economy as those experienced during and after the 2017 Coastal event.

## Materials and methods

### Study area

**Fig 1:**
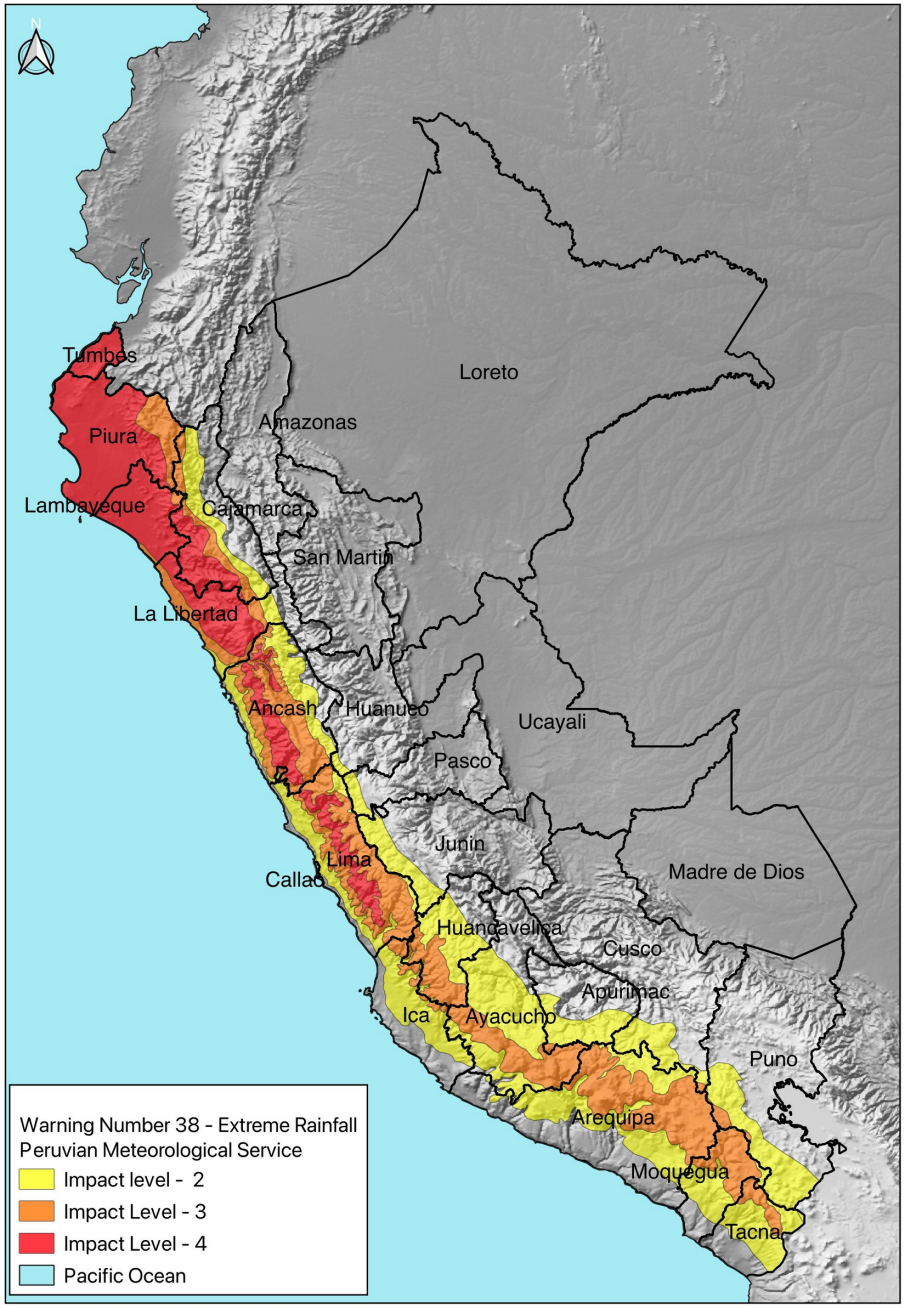
Weather warning for intense rainfall from March 19th to 25th, 2017 (self-elaboration 2021, based on [35] using an open-source natural earth shapefile base).

The 2017 Coastal El Niño strongly affected the coastal regions of Peru, from Tumbes to Ica. (Fig 1). These regions have a large altitude range, from 0 to 3,979 metres above sea level. Peru’s coastal departments (officially called “regions” in Peru) host 60% of the country’s population and most of the country’s largest cities. Annual mean temperatures and precipitation also vary across coastal departments, ranging from 3°C to 32.9°C and 0 to 1,308 mm [31–33] respectively; with limited rainfall in non-El Niño years. Access to water and sanitation is almost universal in this region; however, households not having access to 24-hour water availability on the coast range from 3% to 34.8%, affecting primarily the departments of Ica and Tumbes [34].

### Study design

We assessed the impact of the 2017 Coastal El Niño on four components: climate, economy, health, and policy. For the climatic characterisation, we searched historical climate, atmospheric, and oceanographical records in Peru and literature reviewing the distinctions between ENSO phases and circumstances to define the Coastal El Niño event. For the economic analysis, we used a vector autoregressive (VAR) methodology to determine the economic impact of the 2017 Coastal El Niño event and presented the economic valuation of direct losses by the economic sector measured by the Government.

For the health component of this study, we compiled publicly available national data on cases of mandatory-reporting infectious diseases previously associated with severe weather events. We compared reported infection rates during the 2017 Coastal El Niño with those observed during equivalent periods in the previous five years. For the policy dimension of this case study, to identify potential bottlenecks, limitations and to understand and assess the response of the Peruvian government to the emergency, we conducted in-depth interviews with individuals who led response efforts to the event and reviewed national legislation and policies for disaster emergency response and preparedness at the national, sub-national (regions) and local geopolitical levels (provinces).

### Data collection

#### Climatic characterisation

We used the following observational data: (i) monthly Sea Surface Temperature (SST) data for Puerto Chicama, located in La Libertad department on the northern coast of Peru (7.7°S, 79.4°W) for the 1925–2017 period, from the University of Washington’s Joint Institute for the Study of the Atmosphere and Ocean (JISAO) and the Peruvian Ocean Institute (Instituto del Mar del Perú; IMARPE); and (ii) the monthly Piura river discharge measured at Sanchez Cerro bridge in the city of Piura 1925–2020 from the National Meteorological and Hydrological Service (SENAMHI), JISAO, the National Water Authority (Autoridad Nacional del Agua, ANA), the Chira-Piura Special Project (Proyecto Especial Chira-Piura, PECHP) and the study of Takahashi and Martínez [7]. The monthly Niño 1+2 SST time series is from NOAA OISST v2. The global gridded monthly SST and precipitation data are from NOAA ERSST v5 [36] and GPCP v2.3 [37], respectively.

#### Economy

To determine the economic impact of the 2017 Coastal El Niño event and the economic valuation of direct losses by the economic sector, we used economic data from three national agencies: (i) regional Gross Domestic Product (GDP) data for 1998 from the Institute of National Statistics (INEI), (ii) macroeconomic data related to aggregated GPD (private Investment, private consumption, mining GDP), terms of trade, 1982–1983 total GDP and agriculture and livestock production GDP from the Peruvian Central Bank (BCR) and (iii) ENSO 2017 economic valuation of direct losses of public infrastructure by sector in US$ million from the Special Authority for Reconstruction (RCC) [38–40].

#### Health

To describe the epidemiological context before, during and after the El Niño 2017, we used surveillance data on weekly cases of mandatory-reporting infectious diseases (dengue, chikungunya, zika, leptospirosis, diarrheal disease, respiratory illness, and pneumonia) from 2012 to 2017 from the Peruvian Ministry of Health’s Center for Epidemiology, Disease Control and Prevention (CDC-MINSA).

#### Policy

To collect the data, we conducted in-depth semi-structured interviews with key stakeholders who were involved in the 2017 response. The interviewees were related to the Ministry of Health (MINSA), the Presidency of the Council of Ministers (PCM) and the private industry and provided context and further understanding of the institutional response to the event. These in-depth interviews were tailored to each stakeholder focused on the issues within their jurisdiction. The questions were framed under the main measures applied in the emergency. All interviews were taped-recorded with prior participant consent and took detailed notes. Interviews were transcribed verbatim in Spanish and compared with the notes supporting the transcription process. We completed the assessment by searching and reviewing technical legislation and policy documents regarding disaster response and preparedness from 2010 to 2017.

Details and sources used for this manuscript can be seen in Table 1.

**Table 1:** Summary of data sources.

Source	Data collected
National Centre for Epidemiology Disease Prevention and Control (CDC-Peru), of the Peruvian Ministry of Health (MINSA) / https://www.dge.gob.pe/portalnuevo/	Number of weekly cases of mandatory-reporting conditions (dengue, chikungunya, zika, leptospirosis, diarrheal disease, respiratory illness, and pneumonia).
National Oceanic and Atmospheric Administration (NOAA) / https://www.noaa.gov/	ENSO information
Peruvian Ocean Institute (Instituto del Mar del Perú; IMARPE) / https://www.gob.pe/imarpe	Monthly SST data for Puerto Chicama on the northern coast of Peru (7.7°S, 79.4°W) for the 2003–2017 period.
Proyecto Especial Chira-Piura (PECHP) provided by the Autoridad Nacional del Agua (ANA) / https://www.gob.pe/pechp / https://www.gob.pe/ana	Monthly SST data for 2016–2017 was calculated from the daily values obtained from the website of the PECHP (http://www.chirapiura.gob.pe/). The climatological average was made for each month separately for the period 1981–2010.
Joint Institute for the Study of the Atmosphere and Ocean (JISAO) / http://research.jisao.washington.edu/data_sets/	Monthly SST for Puerto Chicama on the northern coast of Peru (7.7°S, 79.4°W) for the 1925–2002 period Piura river discharge measured in the city of Piura for 1926–1998 (data for 1925 reconstructed by Takahashi)
Special Authority for Reconstruction (RCC) / https://www.rcc.gob.pe/2020/	Economic valuation of direct losses of public infrastructure by sector
Peruvian Central Bank (BCR) / https://www.bcrp.gob.pe/	Macroeconomic data related to aggregated GPD (private Investment, private consumption, mining GDP) Terms of trade 1982–1983 total GDP Agriculture and livestock production GDP from the Peruvian Central Bank
Institute of National Statistics (INEI) / https://www.inei.gob.pe/ / https://www.gob.pe/inei	Regional Gross Domestic Product (GDP) data for 1998
Former Council of Ministries (PCM) Peruvian Ministry of Health (MINSA), Private Industry	In depth interviews with key stakeholders from PCM (2 interviewees), MINSA (2 interviewees) and private industry (2 interviewees)
National Disaster Risk Management System (SINAGERD) (Law 29664) Regulatory Framework for Disaster Risk in Peru	State policy "Disaster Risk Management"—national agreement Law 29664—Law that creates the National Disaster Risk Management System. Supreme Decree 048-2011-PCM approving the regulation of Law 29664. Supreme Decree 111–2012 PCM that provides for the approval of the National Policy as a mandatory policy for the entities of the National Government. Supreme Decree 034–2014 PCM which provides the approval of the National Plan for Disaster Risk Management—PLANAGERD 2014–2021. Law Nº 29230—Works for Taxes

Source: Self-elaboration 2021

### Data analysis

#### Climate characterisation of El Niño events

The climatological mean (1981–2010) of the monthly time series of different climate variables was calculated separately. A monthly series descriptive analysis of the SST of Puerto Chicama, the El Niño 1+2 index, and the Piura River discharge for the El Niño years 1925, 1983, 1998, and 2017 were described with their respective climatological values. We also contrast the El Niño 1+2 index with the reference threshold of 25ºC as an indicator of the onset of heavy rainfall and strong river discharge on the northern coast of Peru. Finally, we compared the spatial patterns of SST and precipitation anomalies in the Tropical Pacific for two El Niño events, 1998 and 2017.

#### Economy

The economic impact of the 2017 Coastal El Niño event was estimated using a Vector Autoregression (VAR) methodology (See S1 Appendix for a detailed explanation). This econometric analysis used macroeconomic and climate variables to isolate the effects of weather anomalies and produce a referential forecast with information as of the 4Q2016 (before the event). Specifically, variables SST and Piura River flow were considered for the economic VAR analysis since they are usually associated with the occurrence of El Niño disasters in the past, and therefore serve two purposes: detection of temperature changes and measure of flooding probability for a representative city (Piura). By comparing forecasted values to the actual performance of the variables mentioned above during 2017, we quantified the “total” impact of the event on GDP growth (both direct and indirect), reflected in macroeconomic data. The economic valuation of direct losses in each economic sector due to El Niño was gathered from official statistics and surveys conducted after the event by the Institute of National Statistics and the RCC.

#### Health

First, we evaluated if, in the Coastal El Niño year (2017), there was a country-wide increase in key weather-sensitive infectious diseases cases under mandatory reporting surveillance (dengue, zika, leptospirosis and diarrheal disease, respiratory illness and pneumonia among children <5 years old), compared to the average of previous, recent and most comparable years (2012–2016). Then, we evaluated whether observed cases of dengue and diarrheal disease, respiratory illness, and pneumonia among children <5 years old increased during and after the onset of Coastal El Niño, both for all of Peru and each of the four northern coastal regions most affected (La Libertad, Lambayeque, Piura, and Tumbes). We compared weekly 2017 cases grouped in five-week periods versus the mean of the equivalent weeks in 2015–2016 and used analysis of variance to determine whether cases increased above expected levels of previous years and, if so, when upon the El Niño onset. Comparisons may slightly underestimate the impact of the 2017 Coastal El Niño, as there was a mild El Niño event between 2015 and 2016, potentially increasing incidence rates in that period.

#### Policy

We followed a semi-structured guide for the interviews to assess the appropriateness of the 2017 Coastal El Niño response at different governmental levels and the effect of the governance structure on this response. By using the international guidance documents on risk management, “Disaster risk management systems analysis: A guide book” from the Food and Agriculture Organization, and the “Information management and communication in emergencies and disasters: Manual for disaster response teams” from the Pan American Health Organization, we assessed and adapted three key capabilities: (i) information management, (ii) rapid adaptation in response to changing conditions, and (iii) coordination between governmental agencies [41, 42]. In addition, we wanted to understand if the existing emergency response protocols, fund allocations and preparedness were appropriate before the event occurred. We read, edited, and coded the information for the analysis based on the three key capabilities. We complemented the analysis with additional information provided by the interviewees (quoted in the results).

### Ethics

The ethical review board of Universidad Peruana Cayetano Heredia reviewed and approved the study (REF: 297-14-19, SIDISI: 103898). All participants who agreed to participate in the study provided verbal consent before being interviewed.

## Results and discussion

### Climate characterisation of El Niño events

**Fig 2:**
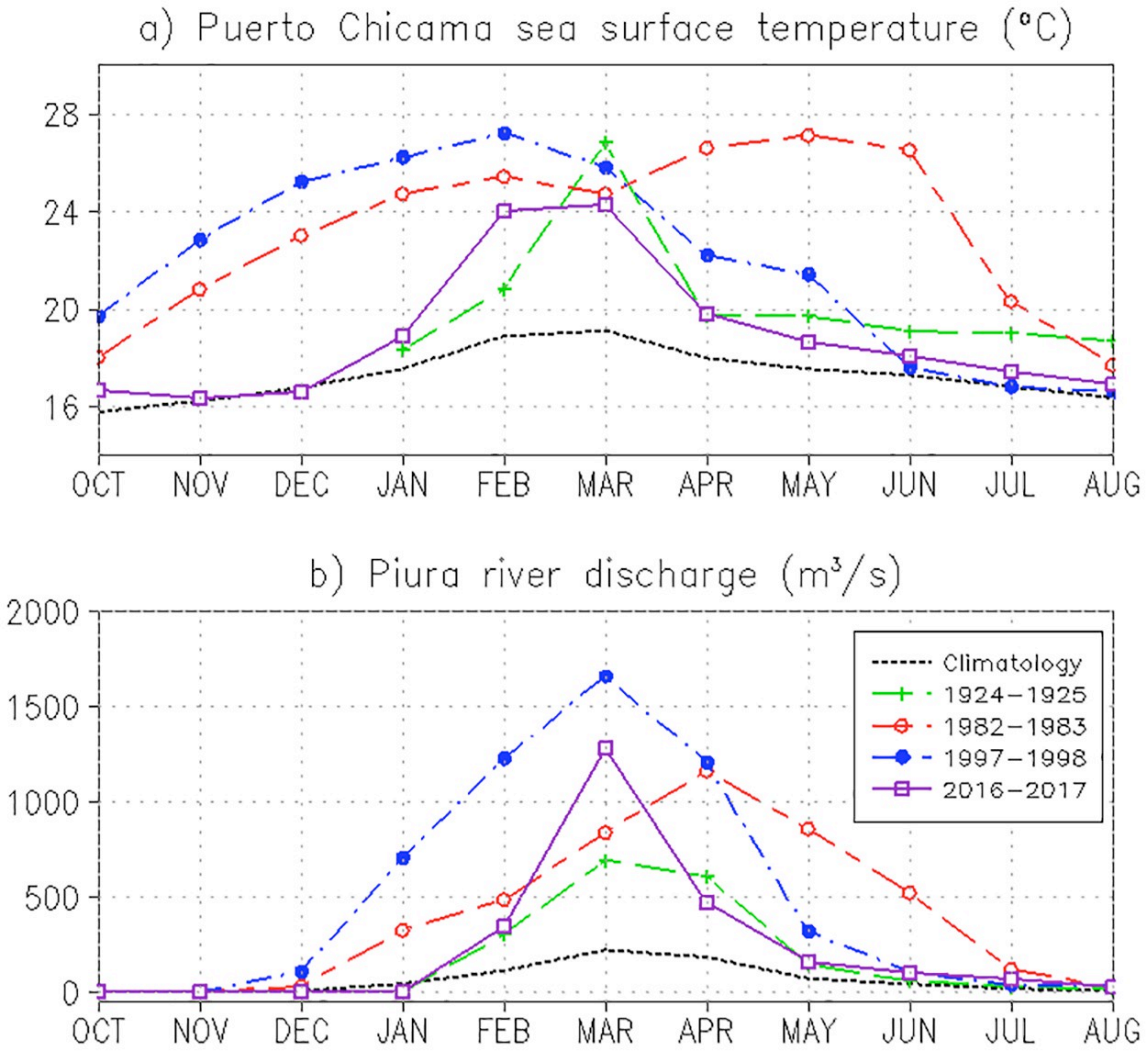
Monthly mean SST (°C) a) at Puerto Chicama, b) in the Niño 1+2 region (reference level of 25°C indicated with a dashed black line), and c) Piura River discharge (m^3^/s) for the 1981–2010 climatology (black dotted) and during the El Niño events of 1925 (green dashed and crosses), 1983 (red short-long dashed and circles), 1997 (blue dot-dashed and filled circles) and 2017 (purple solid and squares). (Self-elaboration 2021 based on NOAA, JISAO, IMARPE, Takahashi and Martínez 2017, ANA, PECHP, SENAMHI).

SST data from Puerto Chicama (Fig 2a) showed that the coastal ocean warming during the extreme El Niño events of 1982–1983 and 1997–1998 was strong but gradual, albeit with somewhat different timing. In the 1982–1983 event, monthly SST was 2.2°C above normal in October and peaked in May 1983 at 9.6°C above normal. In the 1997–1998 event, SST was 3.9°C above normal in October and peaked in January 1998 (8.7°C anomaly). A similar trend was seen in the Niño 1+2 SST Index (Fig 2b), which represented an average over a larger area, so the anomalies have a smaller amplitude, peaking in June 1983 (4.6°C) and December 1998 (4.1°C).

It is important to note that, rather than SST anomalies, it is the absolute SST that matters most for precipitation. The reference threshold of around 25°C can be taken roughly as the minimum SST required to trigger precipitation in northern Peru [43], although, for the Piura River, it is more accurate to use the relationship with the SST gradient [7]. Fig 2b shows that the period when the SST of Niño 1+2 index exceeds 25°C coincides well with the period when the Piura River flow starts increasing, which in the climatological mean corresponds to February-April. For the extreme Niño of 1982–1983 and 1997–1998, the condition of SST > 25°C was met for December-July and November-June, respectively (Fig 2b). However, the highest discharge of the Piura River was observed in January-June and January-May, respectively, with the peaks of the monthly river flow also coinciding with the SST peaks of the Niño 1+2 index.

In contrast, the onset of the 1925 and 2017 Coastal El Niño was abrupt and of shorter duration according to the Puerto Chicama time series SST (Fig 2a), with the peaks (both in absolute SST and anomaly) in March 1925 (7.7°C above normal) and February 2017 (5.2°C). The SST of the El Niño 1+2 index for 2017 showed an excess of 25°C between January and April, starting only one month earlier than the climatological mean. Consistent with this, the timing of the monthly Piura River flow for both 1925 and 2017 is similar to climatology, with the peaks both in March, with values 3 and 6 times the climatological mean, respectively (Fig 2c).

**Fig 3:**
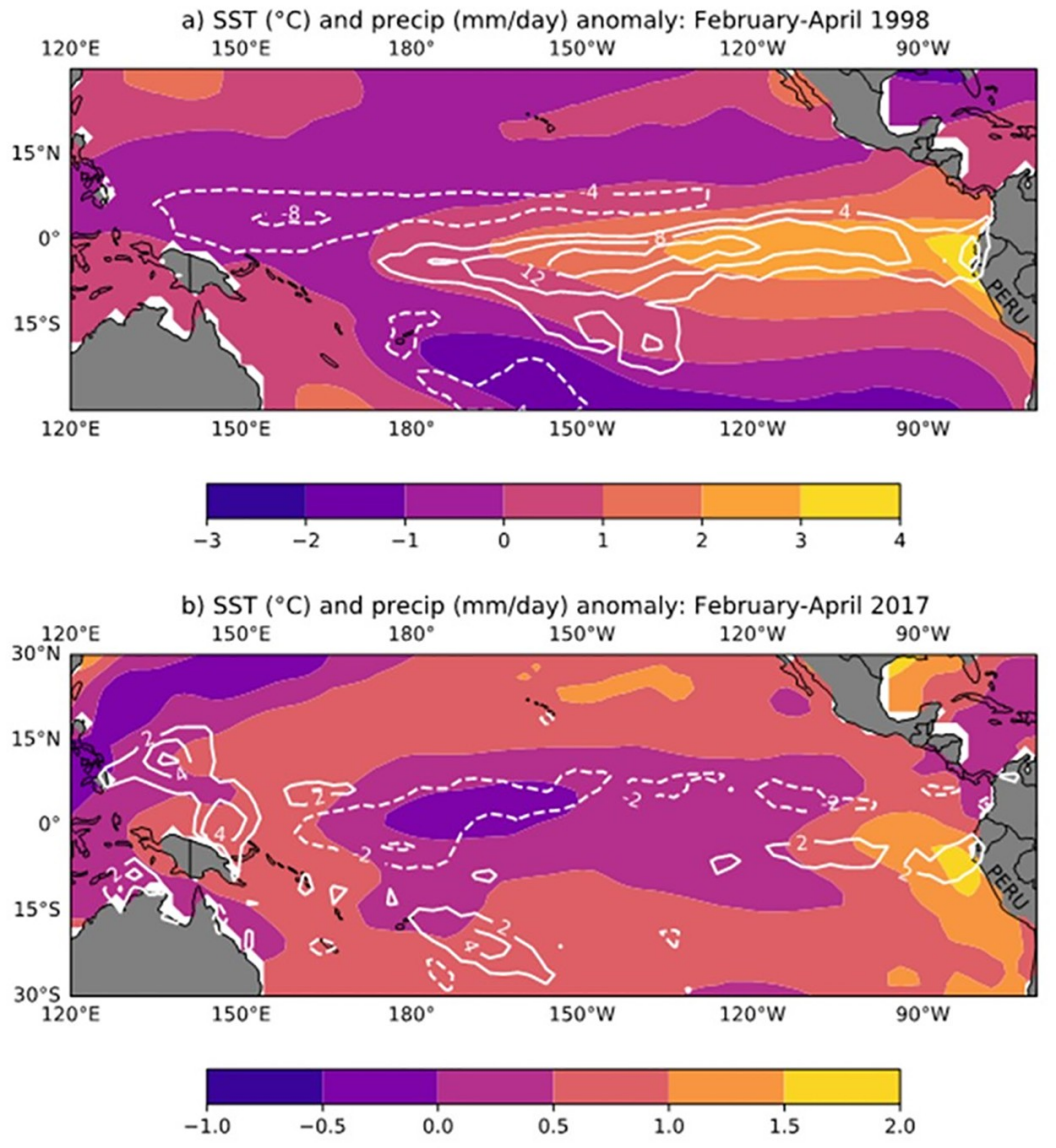
February-April mean SST (shading; °C) and precipitation (contours, solid positive; mm/day) variation across the tropical Pacific basin for two El Niño events: a) extreme ENSO (1998) and b) a Coastal El Niño (2017). There were no warming signals across the central Pacific basin before the Coastal El Niño event (Data from NOAA ERSST v5, GPCP v2.3).

Despite similarities in the impacts on the northern coast of Peru in terms of high SST and heavy rainfall (Fig 3a and 3b), the 1998 global ENSO events and the extreme coastal El Niño events of 2017 had very different signatures in the tropical Pacific Ocean during the February-March high rainfall season. Firstly, the 1998 event was not only more intense along the coast, but its warm and wet anomalies extended along the equator to the central Pacific (Fig 3a). On the other hand, during the 2017 event, the warmest SST anomalies were confined to the eastern end of the Pacific (Fig 3b), with precipitation anomalies of opposite signs north and south of the equator, consistent with the strengthening of the southern branch of the Intertropical Convergence Zone (ITCZ) at the expense of the northern branch. As we will see in the next sections, the intensity and abruptness of this event, despite its short duration, caused important economic losses and public health impacts.

### Economic impacts of coastal El Niño

#### Economic activity

El Niño events of high intensity (such as those registered in 1982–1983 and 1997–1998) are among the climate phenomena events that have recently caused the greatest negative impact on economic activity and living conditions in Peru [44]. In both the 1982–1983 and 1997–1998 events, agriculture was the economic activity that suffered the most in terms of destroyed capital stock and goods produced, affecting mainly small-scale, low-income farmers. Commerce and transportation were other economic sectors with considerable income losses due to the blockages in main highways caused by flooding and mudslides, which isolated several communities for extended periods [23].

In 1983, Peru’s GDP fell by 10%, and estimates suggest that nearly half of the contraction in economic activity was explained by weather conditions related to the 1982–1983 El Niño [45]. The Impacts of the 1998 El Niño were lower: the country’s GDP fell by 0.4%, and agriculture and livestock production stagnated. However, the most affected region was Peru’s northern coast due to mudslides (or *"huaicos"*, *“huaycos”)* that were observed in Tumbes, Piura, Lambayeque La Libertad, and Lima, which affected the economic activity [44].

The northern coast registered a 3% contraction in its GDP level. One of the most affected economic sectors, fishing, recorded a GDP fall of -35.6% in 1998 compared to 1997’s output [46]. According to the Development Bank of Latin America [45], if the El Niño event had not occurred, the 1998 Peruvian GDP would have grown 3% instead of dropping 0.4%. Moreover, the global economic crisis worsened the overall economic performance of Peru during extreme El Niño periods, such as the beginning of the Latin American debt crisis during the 1982–1983 El Niño and the Asian crisis in 1998.

Despite the severity of infrastructure damage, the estimated impact of the 2017 Coastal El Niño on national economic activity–measured in 2017 GDP percentage points lost–was lower than the recorded effect of previous severe El Niño events. This could be explained by the significant diversification of the Peruvian economy in the past 20 years and important innovations in sectors such as agriculture, driven by the increase in international trade and investment [47]. The national production of agricultural products that require higher technological investment and are generally exported was not significantly affected during 2017. These include mango (1.9% growth), avocado (2.5% growth) and asparagus (1.3% growth). In contrast, other products which rely on more traditional agriculture showed negative growth figures in the same year, such as rice (-1.6%), sugar cane (-4.4%) and lemon (-37.9%). This might have been related to the financially stronger and more technologically developed sectors having made greater investments in preparedness systems and assigned greater resources to partially renew damaged areas and promptly resume commercial activities, counteracting climate effects.

Furthermore, the 2017 Coastal El Niño was not an event of a national-level impact but mainly a regional event with an intense, focal impact on the northern coast of Peru. This could also explain why its effects were lower than expected. This region only accounts for 20% of Peru’s population, 16% of the GDP, and 18% of household spending. Regional total GDP fell in the main regions of the northern coast during 1Q2017: Piura (-3.6%), Ancash (-2.6%), La Libertad (-1.1%) and Lambayeque (-0.2%). Particularly, the agriculture and livestock GDP component remained affected in some of these regions throughout 2017: Piura fell by -20.2% and Ancash by -0.2%, while La Libertad and Lambayeque presented small growth figures 0,6% and 0.7%, respectively.

Table 2 shows the expected growth of private investment and private consumption made by the VAR model and the modelled influence that each variable shock had on the difference between the baseline and actual scenarios. For both private investment and private consumption, row (A) shows the expected baseline or variable growth (in percentage change) from 4Q2016. Rows (a), (b), (c) and (d) show the effect attributed to each variable shock on the baseline prediction (in percentage points). By adding up these rows, it is possible to approximate the actual observed growth for each variable during the period. The last column shows the average impact that each variable had during 2017.

**Table 2:** Historical decomposition of economic growth shocks for 2017.

**Private Investment**						
**Shocks**	**4Q2016**	**1Q2017**	**2Q2017**	**3Q2017**	**4Q2017**	**Average 2017**
(A) Baseline scenario	0.7	4.1	5.3	6.5	6.7	5,7
(a) Sea temperature anomaly	-0.1	-0.6	-1.5	-1.2	-0.1	-0.9
(b) Piura River water flow	0.5	-3.5	-4.5	-3.5	-2.9	-3.6
(c) Terms of trade shock	0.1	0.8	1.3	1.4	2.3	1.5
(d) Private investment shock	-6.1	-5.8	-2.3	2.8	-2.6	-2.0
(e) Private consumption shock	0.1	-0.3	-0.9	-0.5	-0.2	-0.5
Actual Private Investment (A) + (a) + (b) +(c) +(d) +(e)	-4.8	-5.3	-2.6	5.5	3.2	0.2
**Private Consumption**						
**Shocks**	**4Q2016**	**1Q2017**	**2Q2017**	**3Q2017**	**4Q2017**	**Average 2017**
(A) Baseline scenario	5.0	4.5	3.8	4.0	3.7	4.0
(a) Sea temperature anomaly	0.0	0.7	-0.6	-1.0	-0.9	-0.5
(b) Piura River water flow	0.0	-0.2	-0.8	-0.8	-0.7	-0.6
(c) Terms of trade shock	0.1	0.2	0.1	0.3	0.6	0.3
(d) Private investment shock	-1.0	-1.8	0.6	-0.1	0.5	-0.2
(e) Private consumption shock	-1.1	-1.2	-0.7	0.4	-0.6	-0.5
Actual Private Consumption (A)–(a)–(b)–(c)–(d)–(e)	3.0	2.2	2.5	2.7	2.6	2.5

All figures are expressed in terms of real per cent growth.

Source: Self-elaboration 2021

Our econometric vector autoregression (VAR) analysis of macroeconomic and climate variables isolated the effects of weather anomalies and used data before the Coastal El Niño (fourth quarter of 2016) to produce a referential 2017 economic activity forecast (baseline). The differences between the baseline forecast and the actual observed data can be decomposed by individual shocks related to the outcome of each variable after the prediction. For example, a private investment shock shows how much of the aggregate forecast error can be explained by the performance of this variable, which in our results is negative, probably due to a loss of business confidence in this period. Similarly, a negative consumption shock can be understood as a loss in consumer confidence, while a positive term of trade shock can be related to higher-than-expected main export prices. Finally, the sea temperature anomalies and the Piura River water flow negative shocks represent how much of the prediction error is explained by the unexpected climatic conditions of the Coastal El Niño.

Our estimates showed that the Coastal El Niño had a negative effect of -4.5 and -1.1 percentage points on private investment growth and private consumption growth, respectively (obtained by addition of rows (a) and (b)). Considering that in 2016 these economic aggregates represented 18% and 64% of GDP, respectively, we can give an approximate aggregate GDP impact by multiplying each negative effect by the individual weight of these indicators on GDP. These results translate into an estimated aggregated negative impact of -1.5 percentage points on 2017 GDP growth. So, GDP growth in 2017 would have reached 4.0% if the Coastal El Niño had not occurred, instead of the officially recorded 2.5% growth.

#### Public infrastructure

Despite the well-known impacts of El Niño-related floods on public infrastructure such as roads, bridges, schools, and healthcare facilities, investments to prevent damage in the most vulnerable areas have remained insufficient. It was no surprise that the intense rainfall and mudslides of the 2017 Coastal El Niño brought about such expected damage to main roads and agricultural areas and floods in urban and rural areas of the northern coast. In total, 1,159 health facilities were affected, of which 32 collapsed, and 39 were declared inhabitable in a context where 1,644,879 people were affected and 505 injured [23].

In addition, 3,703 schools were damaged, 141 collapsed, and 315 were categorised as inhabitable [23]. Transportation infrastructure was severely affected: 493 bridges collapsed, an additional 943 were damaged, 6,428 km of highways and rural roads were destroyed, and an additional 16,1905 km were affected [23]. Infrastructure damage was greater on the northern coast and lesser along the central coast and the highlands. Major flooding took place in three of the largest cities in Peru: Lima, Trujillo, and Piura, severely affecting urban infrastructure. Water and sanitation services collapsed due to ruptures generated by mudflows.

Almost 49,000 housing units were affected or damaged, and streets and sidewalks were severely affected. Vulnerable areas, due to their proximity to riverbanks, were worse off. Repairing all damaged public infrastructure in urban and rural areas and installing prevention infrastructure would cost approximately US$ 7.7 billion, approximately 3,5% of Peru’s GDP and 11% of the total GDP of the five most affected regions (Table 3).

**Table 3:** Estimated costs of reconstruction of public infrastructure damaged by the 2017 Coastal El Niño. Used an average exchange rate of S/3.26 per US$ to transform it from S/ to US$ [40].

Type of infrastructure	Description	Total cost (US$ Million)	% of total
Transportation	Highways, roads, and bridges at the national and local level	2,994	38.8
Education	Public schools’ renewal or reconstruction	819	10.6
Water and sanitation	Renewal of water supply and sewage facilities	626	8.1
Urban roads	Reconstruction of affected roads and sidewalks	455	5.9
Agriculture	Reconstruction of irrigation channels	413	5.4
Health	Renewal or reconstruction of hospitals and small-scale health facilities	413	5.4
Housing	Renewal or relocation and reconstruction of affected houses	342	4.4
Prevention	River defences, reservoirs, and sewage systems	1,650	21.4
Total		7,712	100.0

Required investments to complete such massive reconstruction could take many years, considering Peru’s track record of deficient public investment. Between September 2017 and April 2019, only 27% of the total approved budget related to the reconstruction works was used [48]. Since the start of the reconstruction program, there have been major changes in the legal reconstruction framework. A Government to Government (G2G) agreement with the United Kingdom was recently implemented to accelerate the project’s pipeline and execute the required prevention projects [49].

#### Health assessment

In 2017, the CDC-Peru documented over 68,000 dengue cases, representing Peru’s largest dengue outbreak in history and a two-fold increase from the maximum annual cases observed between 2012 and 2016 (Table 4). The four most battered regions by El Niño, all on the northern coast, accounted for 82% of the cases, despite having only 17% of Peru’s population, and Piura, the epicentre, had 65% of all the dengue cases that year. Mean dengue cases in Piura in 2017, before the El Niño emergency status was declared, were 12% below the mean of the corresponding 2015 and 2016 weeks. In the following five weeks of the event, the mean of cases increased marginally significantly to 60% above the 2015–2016 mean (p = 0.070). Then, in the next five weeks, it rose to 404% above the 2015–2016 mean (p<0.001), remaining increased for 15 additional weeks (For more details, see Table 1 in S2 Appendix).

**Table 4:** Number of annual, countrywide cases of climate-sensitive infectious diseases under mandatory surveillance [50].

Disease	2012	2013	2014	2015	2016	2017	2012–2016 mean ± standard deviation	2017/2016-2012 excess (%)
Dengue	28,505	13,092	17,234	35,817	25,160	68,290	23,962 ± 9,028	185.0%
Zika	-	-	-	-	1,572	6,099	1,572	288.0%
Leptospirosis	1,966	1,889	2,413	2,376	2,062	3,319	2,141 ± 240	55.0%
Diarrhoeal disease, <5 years old	525,811	511,228	487,601	513,975	532,553	499,567	514,234 ± 17,234	-2.9%
Respiratory infections, <5 years old	2,801,593	2,912,228	2,644,474	2,653,659	2,793,147	2,595,350	2,761,0220 ± 112,529	-6.0%
Pneumonia, <5 years old	30,793	30,065	25,895	25,169	26,405	26,112	27,665 ± 2,574	-5.6%

More than 5,000 weekly dengue cases were reported countrywide for five weeks between April and May, with a peak of 7,072 cases in the second week of May (week 18). Weekly cases increased substantially both countrywide and in Piura following the flooding in the city of Piura on March 25^th^ (week 12). While the timing of the peak in cases followed dengue’s typical seasonal patterns, cases in 2017 exceeded by 4.9 standard deviations the 2012–2016 annual mean (Table 4), a highly unlikely event given the annual dengue incidence in those years (probability < 0.00005%). In addition, in 2017, there was a record of 89 deaths due to dengue, 61 of them in Piura. Also, Zika cases increased nearly four-fold in 2017 versus 2016, and most of the increase took place in the coastal regions affected by the Coastal El Niño event.

Reported countrywide leptospirosis cases in 2017 were also 4.9 standard deviations above the 2012–2016 annual mean (probability < 0.00005%), mainly due to a sizable increase between January and June 2017 in Piura and Tumbes [51]. In contrast, the 2017 total cases countrywide of other El Niño-sensitive diseases with shorter incubation periods, such as diarrheal disease, acute respiratory infections, and pneumonia, were lower than the 2012–2016 mean. However, the national total of episodes of diarrheal disease and pneumonia increased in the first five weeks after the El Niño emergency was declared compared to the same weeks in 2015–2016 (p = 0.017 and p<0.001, respectively).

Mean weekly cases of diarrheal disease, acute respiratory infections and pneumonia increased significantly after the El Niño onset in Piura (p = 0.006, p>0.001 and p = 0.004, respectively), in addition to other specific increases in specific regions (See Table 2 in S2 Appendix). The significant increases in diarrheal and respiratory infections observed in the northern regions account for 55% and 94% of the excess cases observed countrywide during the 2017 Coastal El Niño. At the same time, although small, the increase in pneumonia cases represented an early and off-season transmission wave.

### Policy assessment

Peru’s disaster risk management system has a complex structure comprising several actors (Box 1). The 2017 Coastal El Niño event made the gaps in the country’s system preparedness and response structure visible. The public sector stakeholders mentioned that the most relevant evidence gaps were: (i) the inadequate management and dissemination of information to national and sub-national levels and general audiences; (ii) the slow adaptation and allocation of funds in response to the fast-changing conditions of the event and (iii) the managerial changes which lead to slower coordination and response. During the interviews, the need to reduce population vulnerability to environmental hazards by improving territorial and environmental management and preparedness, specifically in high-risk areas, was also mentioned.

**Box 1. Peru’s disaster risk management system.**

Since 2011, the National Disaster Risk Management System (SINAGERD) conformed in the Law 29664 [53] is responsible for identifying and reducing risks, preparing for and responding to disasters-lays down in the national disaster response system, which is comprised of several governmental agencies from all five governmental levels, National (or Central Government), Regional (26 regions), provincial municipalities (196 provinces), district municipalities (1,869 districts) and community governments. The entity responsible for SINAGERD is the Presidency of the Cabinet of Ministries (PCM), which can convene the National Council for Disaster Risk Management, a public organisation responsible for political decision-making and strategic coordination. At the national level, Peru has the National Civil Defence Institute (INDECI), responsible for preparedness and response in the event of a disaster and the National Center for Estimating Prevention and Reduction of Disaster Risk (CENEPRED), responsible for prevention and reconstruction. INDECI and CENEPRED were ascribed to PCM, but this was changed to the Ministry of Defence (MINDEF) in 2016 and 2017, respectively. Furthermore, during the 2017 Coastal El Niño, INDECI assumed the functions of the PCM office associated with disaster risk management and was charged with the technical secretariat of the National Council. Also, considering its relatively large budget, INDECI was effectively the responsible entity in the national response to the 2017 El Niño event [54]. At the regional and municipal levels, the corresponding governments are responsible for all the processes in disaster risk management. In the case of El Niño, the Regional/Local Emergency Operation Centres (COER/COEL) are responsible for monitoring, analysing, and disseminating the information from the National Meteorological and Hydrological Service (SENAMHI), the Regional Health Offices (DIRESA) and the Commission for the National Study of El Niño (ENFEN), as well as from the National Emergency Operation Centre (COEN), led by INDECI. Local governments constitute the first level of response and surveillance in case of disasters through the National Plan for Disaster Risk Management (PLANAGERD), the municipal civil defence groups and the Emergency Centre of Operation (COE). Local governments execute annual budgets in the Reduction of Vulnerability and Attention to Disaster Emergencies Budget Programme PP068 (PREVAED) framework to manage these actions.

Civil society´s engagement and adequate information management are crucial steps in disaster risk reduction. Language barriers and inadequate or untimely information communication may lead to disinformation and confusion during an event. According to all the interviewees, situations where the citizens did not understand the forecast and early warning systems due to language limitations and inappropriate dissemination strategies hindered the appropriate response.

"Despite the warnings from the early warning system, many districts failed to carry out preparedness works and communicate effectively to their residents."

(PCM representative)

Moreover, local governments were not prepared for the event despite available annual preparedness funds. Subnational governments (region and province-level) do not have the capabilities to create an integrated disaster risk plan. Furthermore, the interviewee pointed out many authorities lack the political will to apply for and manage funds, mainly due to the rigidity in spending and a lack of human capacity. Usually, this context leads local governments to prioritise initiatives with short-term immediate results and higher visibility and political impact rather than integrated approaches with more considerable, albeit long-term, public gains.

“Very few mayors and regional presidents used the budget allocated in the previous year to prepare for the phenomenon of El Niño”

(PCM representative)

In addition, by restricting the funding schemes to one year, the central government has substantially limited the possibility of developing integrated approaches needed for this kind of climate event. A PCM representative stated that despite knowing the possibility of an El Niño event, the magnitude of the event surpassed the prevention efforts put in place by the local governments. Once the event was on course, the central government declared a state of emergency to access the emergency funds. Thirteen of the 24 departments of Peru (Piura, Tumbes, Ancash, Junin, Huancavelica, Loreto, among others), corresponding to 109 provinces and 879 districts, were declared to be in a state of emergency.

“States of emergency allow local governments to make use of different budget items and overcome control barriers established by the comptroller to speed up the spending required by the emergency.”

(PCM representative)

A PCM representative mentioned that changes in the government structure in December 2016 led to slower bilateral coordination and created bottlenecks in the initial emergency response. This led to a centralised response, where the central government coordinated directly with the National Emergency Operation Centre (COEN), while the ministers of the different portfolios were sent to the regions to coordinate directly with the Regional Emergency Operation Centres (COER). Efficient and effective coordination and communication among all sectors were slow, but sending national representatives allowed faster decision-making and coordination between the municipalities, armed forces, police, firefighters, regional health, agriculture, transportation and communications directorates and private companies.

“There were cases of critical patients, which require immediate transfer, who were mobilised thanks to coordination with the armed forces”

(MINSA representative)

The disaster risk response drove local stakeholders (private companies, NGOs, and the general population) to try and bridge some of the governmental gaps. They donated “goods” during the emergency and were involved in the early reconstruction effort through the mechanism of “works for taxes”, led by the “Pro Inversion” programme of the central government [52].

“An alliance was made between other companies and a conversation with the regional government was made and they presented a list of priority works, and after a brief discussion, we decided to collaborate in the reconstruction of the bridge”

(Private industry)

A clear example of the existing gaps could be seen in managing supplies (water and food) and shelter for the displaced people. The response to re-building the lost infrastructure was slow and inexistent in some areas. Health services could not cope with the increased flow of patients from other flooded areas. Health staff initially turned away patients because of insufficient personnel to treat them or lack of identification cards and public insurance (a requirement for attendance in health establishments). Many health workers lost their homes and were themselves displaced.

“Many health establishments denied care to people because they did not have an ID. In the same way, there were attention problems with social security because in the absence of the identity document they could not be registered”

(MINSA representative).

In addition, the existing health system did not allow for access to health care treatment in camps for displaced populations. In these camps, people had to deal with additional difficulties concerning violence, mental health, reproductive health, and access to care for underlying conditions.

“Many patients with cancer, tuberculosis or HIV lost their medicines and had no access to new ones.”

(MINSA representative)

The last topic that surfaced during the interviews was reducing risks in illegal housing, pointing toward the need for local-level risk evaluations, particularly in vulnerable populations. In recent years, urban expansion has continued to grow disproportionately and often informally, with the most disadvantaged populations occupying the land in dangerous and marginal areas of the cities that have an increased risk of landslides and flooding.

“There are many people who are exposed to risks generated by the environment. On the central coast many people occupy unsuitable spaces, but with the approval of the municipality… (…) The municipalities do not always carry out prevention work in high-risk areas, many wait for an emergency to occur before acting.”

(MINSA representative)

## Conclusions

The 2017 Coastal El Niño event was intense and abrupt, with a rapid onset. It did not correspond to a warm ENSO phase but coincided with neutral to cool ENSO conditions and was confined to the far-eastern Pacific. Because of this intensity and abruptness, climate models used for projections could present biases, particularly in the far-eastern Pacific, which should be considered with care. Despite the short duration of this event, restricted to February-April, a ten-fold increase in heavy rainfall and river flooding did incur important economic losses and health impacts, similar to those of the 1983 and 1998 severe warm ENSO (El Niño) events.

The econometric analysis based on macroeconomic variables and sea temperature anomalies showed that the Coastal El Niño event impacted more than just infrastructure, harming private consumption and private investment, with an aggregate negative impact on GDP growth of -1.5 percentage points in 2017. Higher event frequency than the normal 3–7 year cycle would have a dire effect on Peruvian economics and the country’s ability to mitigate the potential impacts, prepare accordingly and bounce back.

On the other hand, public health impacts with increases in infectious and vector-borne diseases in disaster settings are not unexpected during El Niño events. The health assessment shows that CDC-Peru recorded the largest dengue outbreak ever, with 76,093 confirmed cases, 68,630 (90% of reported cases) in the 2017 Coastal El Niño-affected areas. The analysis also displays an increase in reported leptospirosis cases in Piura, one of the regions most affected by extreme weather. Other immediate impacts on health outcomes, particularly on infectious diseases, may have been difficult to assess with surveillance data due to the interruption of healthcare services and disease reporting during the most severe weeks of the event.

The 2017 Coastal El Niño event also highlighted the challenges in Peru’s disaster response system. It showcased the gaps and potential failures in the health system’s structure and function and the information management and dissemination system. Furthermore, it portrayed how the state responded under the pressure of a disaster situation to protect disenfranchised and vulnerable populations. Preparedness is a key topic, particularly for the different climate change scenarios and their effect on extreme weather events like ENSO or Coastal El Niño events.

Based on the findings, we conclude that the necessary improvements to Peru’s disaster preparedness and response under all climate change scenarios should span a broad set of issues at different geopolitical scales and impacts tailored to the diversity of vulnerabilities. From territorial zoning, strategic health facility placement in the Regions, and versatile information and communication strategies to governance structures and financial mechanisms. Moreover, since the frequency of ENSO events will likely increase and intensify with unabated greenhouse gas emissions [12, 16], there is a need to improve territorial and environmental management and preparedness. This can be addressed through local-level climate change risk assessments (i.e., city level, community level) with a framework of auditable indicators and ensure the assignment of a focal point person at the local level that manages adaptation and mitigation plans, response and preparedness, communication, strategies, and effective budget allocation. Cooperation mechanisms across local, regional, central government and private sectors should be active in the long term and not only during the emergency response. These issues will be critical to the country’s ability to anticipate and mitigate future disasters.

## Supporting information

S1 Appendix Economic activity impact estimation methodology. (DOCX)

S2 Appendix Selected health cases in Peru (2015–2017). (DOCX)
