# Supplementary material for: Reflections on the impact and response to the Peruvian 2017 Coastal El Niño event: Looking to the past to prepare for the future
Source: PLoS One. 2023 Sep 26;18(9):e0290767. doi: 10.1371/journal.pone.0290767 (PMC10522041; doi:10.1371/journal.pone.0290767)
Supplement: S1 Appendix — (DOCX) [file pone.0290767.s001.docx]

**S1 Appendix. Economic activity impact estimation methodology**

The economic impact of the 2017 Coastal ENSO event is estimated using a Vector Autoregression (VAR) methodology based on macroeconomic variables and two proxy variables for climatic conditions.

In its simplest form, VAR analysis involves performing Ordinary Least Squares (OLS) regressions in a multivariate form, explaining each variable in terms of its lagged (past) values and the lagged values of the other variables in the system. The system variables are contained in the y*_t_* vector. Each equation has a vector error term *e_t_* which represents the unexplained section of the process. For this analysis, we used a linear representation which involved no constant term and only one lag (*y_t_*_-1_) of the vector of variables:

y*_t_*=A_1_*y_t_*_-1_+*e_t_*

Where y*_t_* and *e_t_* are vectors with *n*×1 dimension (*n* being the number of variables) and A_1_ is a coefficient matrix of *n*×*n* dimension.

We use five main variables for the estimation: sea temperature anomalies, Piura River water flow, terms of trade, private investment, and private consumption, each with a lag order of one period. Also, mining GDP is considered a control variable. We use quarterly data for the 2Q1982 to 1Q2018 period, accounting for 144 observations.

Two variables are used as proxies for climatic conditions: (i) the sea temperature anomaly variable and (ii) the Piura River water flow. The use of these two variables is relevant, as the 2017 Coastal El Niño does not present an important rise in sea temperature (as opposed to past El Niño events) but does present historically high river flow indicators, reflected in the damages caused by flooding.

**Table 1.** Variables and sources for economic analysis (Self-elaboration 2021)

| **Variables** | **Description** | **Source** | **Period Covered by Data** |
| --- | --- | --- | --- |
| Private investment | Annual real percentage change | Peruvian Central Bank | 2Q1982-1Q2018 |
| Private consumption | Annual real percentage change | Peruvian Central Bank | 2Q1982-1Q2018 |
| Terms of trade | Annual real percentage change | Peruvian Central Bank | 2Q1982-1Q2018 |
| Sea surface temperature anomaly for the Niño 1+2 area | Temperature difference with respect to the historical average of each month | National Oceanic and Atmospheric Administration | 2Q1982-1Q2018 |
| Piura River water flow | Average cubic millimetres per second (m3/s) | National Water Authority | 2Q1982-1Q2018 |
| Mining GDP | Annual real percentage change | Peruvian Central Bank | 2Q1982-1Q2018 |

After estimating the VAR equation, we used its coefficients to compare a naïve forecast with information before the event and the real economic results after the event. To do this, we estimated a baseline scenario of the expected performance of the two main components of total GDP (private investment and private consumption) for the 2017 period, made with information up to 4Q2016 (before the event occurred), by using the lagged values of the other explaining variables. This resulted in a forecasted quarterly trajectory for 2017 of private investment and private consumption.

Afterwards, we could compare the expected values of the variables mentioned above to their actual performance and calculate the contribution that each variable in the model had to the actual results for private investment and private consumption. The contribution of each variable in the observed scenario is represented by the name “shocks”, and this process is called Historical Decomposition. By comparing the expected values to the actual performance of the variables mentioned above, we were able to quantify the “total” impact of the event on GDP growth (both direct and indirect), reflected in macroeconomic data.

The inclusion of the temperature anomaly in the model isolated the effect of extreme climatic changes on economic growth performance from other exogenous shocks. This approach allowed us to estimate a GDP forecast dependent exclusively on the information before the event (fourth quarter of 2016) and to compare it with the actual observed GDP after the event.

Finally, we added the individual effects of the two climatic variables in each of the two main components of total GDP (private investment and private consumption) and obtained a consolidated effect for each component (E1 and E2, respectively). With these consolidated climate effects, we can give an approximate aggregate GDP impact by multiplying each effect with the individual weight of these indicators on GDP.

Agreggate GDP effect= E_1_ X GDP ^priv.consump.weight^ + E_2_ X GDP ^priv.investment.weight^

Considering that in 2016 private consumption and private investment represented 18% and 64% of GDP respectively, we could translate these impacts into an estimated aggregate negative impact on 2017 national GDP growth.
