# Supplementary material for: Reflections on the impact and response to the Peruvian 2017 Coastal El Niño event: Looking to the past to prepare for the future
Source: PLoS One. 2023 Sep 26;18(9):e0290767. doi: 10.1371/journal.pone.0290767 (PMC10522041; doi:10.1371/journal.pone.0290767)
Supplement: S2 Appendix — (DOCX) [file pone.0290767.s002.docx]

**S2 Appendix. Selected health cases in Peru (2015 - 2017)**

**Table 1.** Weekly dengue cases 2015-2017 (CDC Peru, n.d.)

|  | **Absolute number of weekly dengue cases** | | | | | | |  | **Weekly 2017 dengue cases, relative to each year's** | | | | | | |
| --- | --- | --- | --- | --- | --- | --- | --- | --- | --- | --- | --- | --- | --- | --- | --- |
| **Week** | **Peru** | | |  | **Piura** | | |  | **Peru** | | **5-week Mean** |  | **Piura** | | **5-week Mean** |
|  | **2015** | **2016** | **2017** |  | **2015** | **2016** | **2017** |  | **2015** | **2016** |  |  | **2015** | **2016** |  |
| 1 | 272 | 307 | 227 |  | 19 | 14 | 45 |  | 83.5 | 73.9 |  |  | 236.8 | 321.4 |  |
| 2 | 289 | 417 | 250 |  | 32 | 44 | 47 |  | 86.5 | 60.0 |  |  | 146.9 | 106.8 |  |
| 3 | 327 | 451 | 263 |  | 71 | 58 | 48 |  | 80.4 | 58.3 | 75.4 |  | 67.6 | 82.8 | 125.1 |
| 4 | 333 | 473 | 279 |  | 73 | 110 | 59 |  | 83.8 | 59.0 |  |  | 80.8 | 53.6 |  |
| 5 | 375 | 599 | 388 |  | 92 | 122 | 81 |  | 103.5 | 64.8 |  |  | 88.0 | 66.4 |  |
| 6 | 393 | 766 | 541 |  | 130 | 167 | 125 |  | 137.7 | 70.6 |  |  | 96.2 | 74.9 |  |
| 7 | 515 | 769 | 611 |  | 146 | 197 | 174 |  | 118.6 | 79.5 |  |  | 119.2 | 88.3 |  |
| 8 | 519 | 822 | 547 |  | 124 | 210 | 251 |  | 105.4 | 66.5 | 106.1 |  | 202.4 | 119.5 | 153.7 |
| 9 | 596 | 895 | 732 |  | 180 | 206 | 355 |  | 122.8 | 81.8 |  |  | 197.2 | 172.3 |  |
| 10 | 636 | 933 | 1 051 |  | 240 | 278 | 602 |  | 165.3 | 112.6 |  |  | 250.8 | 216.5 |  |
| 11 | 683 | 891 | 1 345 |  | 270 | 194 | 799 |  | 196.9 | 151.0 |  |  | 295.9 | 411.9 |  |
| 12 | 727 | 729 | 1 841 |  | 312 | 158 | 1 105 |  | 253.2 | 252.5 |  |  | 354.2 | 699.4 |  |
| 13 | 702 | 879 | 2 629 |  | 382 | 213 | 1 671 |  | 374.5 | 299.1 | 294.7 |  | 437.4 | 784.5 | 574.3 |
| 14 | 1 108 | 1 138 | 3 936 |  | 726 | 291 | 2 643 |  | 355.2 | 345.9 |  |  | 364.0 | 908.2 |  |
| 15 | 1 421 | 1 200 | 4 678 |  | 997 | 306 | 3 482 |  | 329.2 | 389.8 |  |  | 349.2 | 1 137.9 |  |
| 16 | 1 687 | 1 181 | 6 042 |  | 1 157 | 375 | 4 590 |  | 358.2 | 511.6 |  |  | 396.7 | 1 224.0 |  |
| 17 | 1 725 | 1 060 | 5 816 |  | 1 154 | 344 | 4 316 |  | 337.2 | 548.7 |  |  | 374.0 | 1 254.7 |  |
| 18 | 2 092 | 1 120 | 7 072 |  | 1 414 | 411 | 5 645 |  | 338.0 | 631.4 | 424.2 |  | 399.2 | 1 373.5 | 765.2 |
| 19 | 2 052 | 1 130 | 5 729 |  | 1 380 | 370 | 4 388 |  | 279.2 | 507.0 |  |  | 318.0 | 1 185.9 |  |
| 20 | 2 034 | 1 049 | 5 054 |  | 1 434 | 400 | 3 522 |  | 248.5 | 481.8 |  |  | 245.6 | 880.5 |  |
| 21 | 2 033 | 877 | 3 653 |  | 1 452 | 407 | 2 372 |  | 179.7 | 416.5 |  |  | 163.4 | 582.8 |  |
| 22 | 2 099 | 821 | 3 039 |  | 1 467 | 377 | 1 805 |  | 144.8 | 370.2 |  |  | 123.0 | 478.8 |  |
| 23 | 1 981 | 677 | 2 499 |  | 1 302 | 308 | 1 460 |  | 126.1 | 369.1 | 238.6 |  | 112.1 | 474.0 | 285.9 |
| 24 | 1 749 | 641 | 1 871 |  | 1 100 | 298 | 1 124 |  | 107.0 | 291.9 |  |  | 102.2 | 377.2 |  |
| 25 | 1 371 | 480 | 1 353 |  | 861 | 226 | 797 |  | 98.7 | 281.9 |  |  | 92.6 | 352.7 |  |
| 26 | 1 071 | 356 | 940 |  | 657 | 171 | 575 |  | 87.8 | 264.0 |  |  | 87.5 | 336.3 |  |
| 27 | 915 | 337 | 683 |  | 544 | 176 | 382 |  | 74.6 | 202.7 |  |  | 70.2 | 217.0 |  |
| 28 | 638 | 261 | 588 |  | 330 | 147 | 311 |  | 92.2 | 225.3 | 155.9 |  | 94.2 | 211.6 | 178.2 |
| 29 | 446 | 192 | 458 |  | 218 | 93 | 279 |  | 102.7 | 238.5 |  |  | 128.0 | 300.0 |  |
| 30 | 381 | 168 | 316 |  | 190 | 80 | 190 |  | 82.9 | 188.1 |  |  | 100.0 | 237.5 |  |
| 31 | 341 | 195 | 336 |  | 145 | 104 | 169 |  | 98.5 | 172.3 |  |  | 116.6 | 162.5 |  |
| 32 | 318 | 181 | 214 |  | 163 | 84 | 117 |  | 67.3 | 118.2 |  |  | 71.8 | 139.3 |  |
| 33 | 292 | 128 | 154 |  | 145 | 61 | 70 |  | 52.7 | 120.3 | 105.3 |  | 48.3 | 114.8 | 105.1 |
| 34 | 248 | 84 | 157 |  | 132 | 35 | 75 |  | 63.3 | 186.9 |  |  | 56.8 | 214.3 |  |
| 35 | 323 | 109 | 141 |  | 170 | 54 | 52 |  | 43.7 | 129.4 |  |  | 30.6 | 96.3 |  |
| 36 | 218 | 100 | 121 |  | 104 | 41 | 53 |  | 55.5 | 121.0 |  |  | 51.0 | 129.3 |  |
| 37 | 208 | 110 | 139 |  | 106 | 41 | 49 |  | 66.8 | 126.4 |  |  | 46.2 | 119.5 |  |
| 38 | 200 | 125 | 140 |  | 85 | 40 | 49 |  | 70.0 | 112.0 | 91.3 |  | 57.6 | 122.5 | 81.7 |
| 39 | 241 | 122 | 156 |  | 90 | 41 | 39 |  | 64.7 | 127.9 |  |  | 43.3 | 95.1 |  |
| 40 | 210 | 156 | 151 |  | 58 | 50 | 41 |  | 71.9 | 96.8 |  |  | 70.7 | 82.0 |  |
| 41 | 206 | 202 | 151 |  | 58 | 32 | 35 |  | 73.3 | 74.8 |  |  | 60.3 | 109.4 |  |
| 42 | 167 | 207 | 171 |  | 53 | 30 | 38 |  | 102.4 | 82.6 |  |  | 71.7 | 126.7 |  |
| 43 | 143 | 129 | 122 |  | 37 | 31 | 26 |  | 85.3 | 94.6 | 95.4 |  | 70.3 | 83.9 | 88.7 |
| 44 | 134 | 183 | 148 |  | 38 | 23 | 24 |  | 110.4 | 80.9 |  |  | 63.2 | 104.3 |  |
| 45 | 131 | 214 | 203 |  | 25 | 33 | 28 |  | 155.0 | 94.9 |  |  | 112.0 | 84.8 |  |
| 46 | 135 | 210 | 217 |  | 25 | 17 | 20 |  | 160.7 | 103.3 |  |  | 80.0 | 117.6 |  |
| 47 | 98 | 245 | 179 |  | 27 | 28 | 25 |  | 182.7 | 73.1 |  |  | 92.6 | 89.3 |  |
| 48 | 132 | 197 | 218 |  | 20 | 23 | 25 |  | 165.2 | 110.7 | 122.2 |  | 125.0 | 108.7 | 98.6 |
| 49 | 190 | 188 | 222 |  | 28 | 21 | 28 |  | 116.8 | 118.1 |  |  | 100.0 | 133.3 |  |
| 50 | 229 | 165 | 184 |  | 36 | 26 | 21 |  | 80.3 | 111.5 |  |  | 58.3 | 80.8 |  |
| 51 | 231 | 136 | 171 |  | 22 | 26 | 25 |  | 74.0 | 125.7 |  |  | 113.6 | 96.2 |  |
| 52 | 252 | 155 | 164 |  | 22 | 18 | 23 |  | 65.1 | 105.8 |  |  | 104.5 | 127.8 |  |
| Total | 35 817 | 25 160 | 68 290 |  | 20 043 | 7 610 | 44 275 |  |  |  |  |  |  |  |  |

**Table 2.** Mean increase in weekly cases in the five weeks after the El Niño emergency (CDC Peru, n.d.)

| **Region** |  | **Diarrheal disease** | **Respiratory infections** | **Pneumonia** |
| --- | --- | --- | --- | --- |
| La Libertad | Cases | 61.9 | 264.4 | 7.1 |
|  | p value | 0.233 | 0.101 | 0.026 |
| Lambayeque | Cases | 279.4 | 162.8 | 3.3 |
|  | p value | <0.001 | 0.386 | 0.403 |
| Piura | Cases | 182.0 | 904.0 | 19.4 |
|  | p value | 0.006 | <0.001 | 0.004 |
| Tumbes | Cases | 22.5 | 76.8 | 7.2 |
|  | p value | 0.192 | 0.193 | 0.002 |
| Peru | Cases | 840.4 | 1 238.2 | 134.5 |
|  | p value | 0.017 | 0.625 | <0.001 |
|  |  | 461.4 | 1 168.4 | 33.7 |
|  |  | 55% | 94% | 25% |
